# LncRNA CRNDE attenuates chemoresistance in gastric cancer via SRSF6-regulated alternative splicing of PICALM

**DOI:** 10.1186/s12943-020-01299-y

**Published:** 2021-01-04

**Authors:** Feifei Zhang, Hui Wang, Jiang Yu, Xueqing Yao, Shibin Yang, Weidong Li, Lijun Xu, Liang Zhao

**Affiliations:** 1 Department of Pathology, Nanfang Hospital, Southern Medical University, Guangzhou, China; 2 Department of Pathology, Guangdong Provincial Key Laboratory of Molecular Oncologic Pathology, School of Basic Medical Sciences, Southern Medical University, Guangzhou, China; 3 Department of Medical Oncology, Affiliated Tumour Hospital of Guangzhou Medical University, Guangzhou, China; 4 Department of General Surgery, Nanfang Hospital, Southern Medical University, Guangdong Provincial Engineering Technology Research Center of Minimally Invasive Surgery, Guangzhou, China; 5 Department of General Surgery, Guangdong General Hospital, Guangdong Academy of Medical Science, Guangzhou, China; 6 Gastrointestinal Surgical Center, The First Affiliated Hospital of Sun Yat-Sen University, Guangzhou, China

**Keywords:** Gastric cancer, Long noncoding RNA CRNDE, Chemoresistant, Autophagy, Protein splicing

## Abstract

De novo and acquired resistance, which are mainly mediated by genetic alterations, are barriers to effective routine chemotherapy. However, the mechanisms underlying gastric cancer (GC) resistance to chemotherapy are still unclear. We showed that the long noncoding RNA CRNDE was related to the chemosensitivity of GC in clinical samples and a PDX model. CRNDE was decreased and inhibited autophagy flux in chemoresistant GC cells. CRNDE directly bound to splicing protein SRSF6 to reduce its protein stability and thus regulate alternative splicing (AS) events. We determined that SRSF6 regulated the PICALM exon 14 skip splice variant and triggered a significant S-to-L isoform switch, which contributed to the expression of the long isoform of PICALM (encoding PICALML). Collectively, our findings reveal the key role of CRNDE in autophagy regulation, highlighting the significance of CRNDE as a potential prognostic marker and therapeutic target against chemoresistance in GC.

**Supplementary Information:**

The online version contains supplementary material available at 10.1186/s12943-020-01299-y.

## Main text

Gastric cancer (GC) is a serious health problem and the second leading cause of cancer-related death worldwide [1]. Most patients with advanced GC initially respond to combined chemotherapy [2]. Patients eventually experience tumor recurrence due to drug resistance [3]. Hence, there is an urgent need for new strategies to reverse drug resistance, and understanding the mechanism of cancer cell resistance to chemotherapy is an important step to predict or overcome drug resistance. Recent studies have shown that autophagy plays a major role in regulating the response to chemotherapy [4]. Increasing evidence shows that autophagy can promote the survival of cancer cells and increase drug resistance under chemotherapy pressure [5]. However, under chemotherapy pressure, the key regulatory mechanism leading to the increase in autophagic flux and autophagic degradation in cancer cells is still unclear. To date, studies of lncRNAs regulating chemotherapy resistance in gastric cancer through autophagy are still very limited [6]. The lncRNA CRNDE (colorectal neoplasia differentially expressed) is abnormally expressed in various cancers and plays important roles in proliferation, apoptosis, metastasis, chemoresistance and radioresistance [7]. However, the potential effect of CRNDE on autophagy in GC cells and whether it affects the efficacy of chemotherapy remain unclear. SRSF6 belongs to the splicing factor SR family [8], participates in mRNA splicing and may play a role in the determination of alternative splicing [9, 10]. In this study, SRSF6, as a binding protein of CRNDE, was involved in the regulation of cell chemotherapy resistance. Further functional studies showed that CRNDE binds to serine and arginine rich splicing factor 6 (SRSF6) and reduces its stability, which in turn reduces alternative splicing of PICALM. Our findings provide insights into the significance of CRNDE in predicting the benefits of oxaliplatin and 5-FU adjuvant chemotherapy in patients with GC.

### Low CRNDE suppresses the response to 5-FU/oxaliplatin-based chemotherapy via enhancing autophagy flux in GC patients and the PDX model

**Fig. 1:**
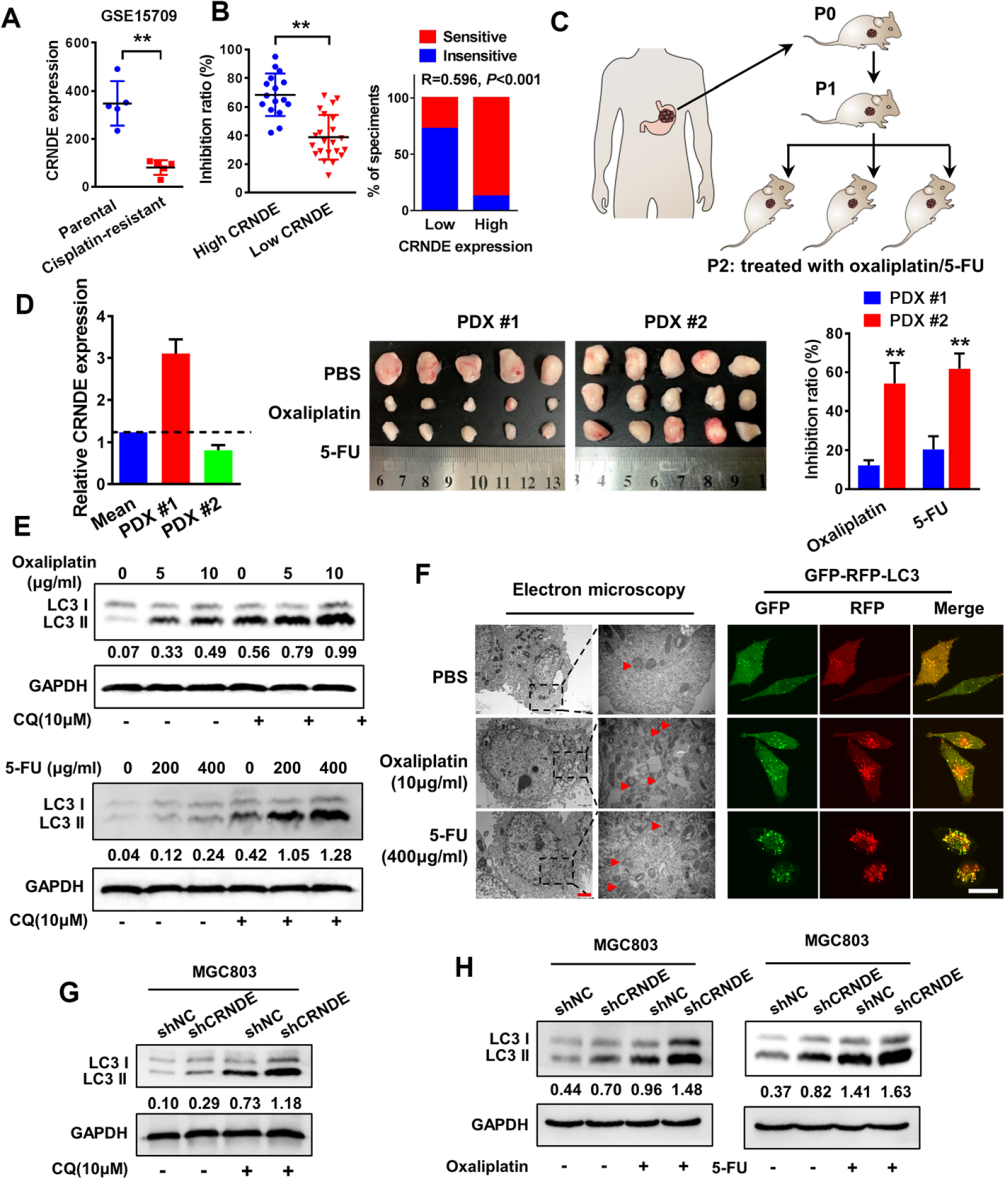
Low CRNDE suppresses the response to 5-FU/oxaliplatin-based chemotherapy via enhancing autophagy flux in GC patients and the PDX model. **a** The expression of CRNDE in cisplatin-resistant gastric cancer cell lines and their parental cells were analyzed by GSEA database. **b** CCK8 assay was used to evaluate the relationship between CRNDE expression and chemosensitivity in 38 cases of GC specimens. **c** Schematic diagram of PDX model of gastric cancer. **d** RT-PCR analysis of the expression level of CRNDE in two cases of PDX models. PDX #1 and #2 tumors were subcutaneously injected into the NOD/SCID mice. The mice were treated with oxaliplatin and 5-FU when the tumor volume reached 50 to 100 mm^3^. The dose of oxaliplatin was 10 mg / kg mice, and 5-FU 50 mg/kg mice were injected intraperitoneally every 3 days for 24 days. The tumor image and tumor inhibition rate (compared to PBS group) of each indicated group are shown (*n*=5). **e** Western blot was performed in MGC803 cells treated with different concentrations of oxaliplatin, 5-FU or CQ. **f** The autophagy and autophagy flow in MGC803 cells was observed by transmission electron microscopy and confocal microscopy when treated with 10 μg/ml oxaliplatin or 400 μg/ml 5-FU. Scale bars, 2 μm (TEM) and 10 μm (confocal microscopy). **g** Western blot of autophagy marker LC3II expression levels in MGC803 cells transfected with shCRNDE plasmid in the presence of CQ. **h** Western blot of autophagy marker LC3II expression levels in MGC803 cells transfected with shCRNDE plasmid in the presence of 10 μg/ml oxaliplatin or 400 μg/ml 5-FU. Student’s *t*-test; mean ± SD; the asterisk (**) indicates *P* < 0.01

We first analyzed the expression level and subcellular localization of CRNDE in 86 archived paraffin-embedded GC tissues by in situ hybridization (ISH). The results showed that CRNDE was expressed in both the cytoplasm and nucleus and its expression was not statistically significant between CRC and normal mucosa (*P*=0.459; Fig. S1A). To investigate the role of CRNDE in GC, we used the bioinformatics database KM-Plotter (*http://kmplot.com/analysis/*) to analyze the relationship between CRNDE and the prognosis of patients with gastric cancer. The results showed that there was no correlation between CRNDE expression and the overall survival of patients with gastric cancer or patients under 5-FU-based chemotherapy regimens (*P* > 0.05), but the prognosis of patients with high CRNDE expression was significantly better than that of patients with low CRNDE expression under other chemotherapeutic drug-based chemotherapy regimens (*P* < 0.05, Fig. S1B). Analysis of the GSO database revealed that CRNDE expression was significantly lower in cisplatin-resistant gastric cancer cells than in their parental cells (*P* < 0.01, Fig. 1a). To explore the role of CRNDE in the chemotherapy response of gastric cancer, we first detected the expression of CRNDE in 38 fresh tissues of gastric cancer by RT-PCR. Sixteen and eighteen samples were defined as the high CRNDE expression group and low CRNDE expression group according to the median expression level, respectively. The drug sensitivity of primary cells extracted from 38 cases of gastric cancer was detected by CCK8 assays. The results showed that the group with high expression of CRNDE had high sensitivity to chemotherapeutic drugs, while the group with low expression of CRNDE was not sensitive to chemotherapeutic drugs, suggesting that the expression of CRNDE is positively correlated with the sensitivity of gastric cancer tissue to chemotherapeutic drugs (*P* < 0.01, Fig. 1b). Next, we tested the chemosensitivity of two GC-derived types of tissues (one with high expression of CRNDE and one with low expression of CRNDE) by the PDX model (Fig. 1c&S1C). The results showed that the chemosensitivity of PDX mice with high expression of CRNDE to oxaliplatin and 5-FU was higher than that of the CRNDE low expression group, suggesting that CRNDE could be related to the chemosensitivity of gastric cancer patients (Fig. 1d). The sensitivity of MGC803 cells to oxaliplatin and 5-FU was increased after overexpression of CRNDE, while the sensitivity of MGC803 cells to oxaliplatin and 5-FU was significantly decreased by silencing the expression of CRNDE (Fig. S1D). We further investigated whether inhibition of CRNDE expression could reduce the sensitivity of MGC803 cells to chemotherapy. The results showed that compared with the mice without chemotherapy, the mice treated with oxaliplatin and 5-FU had significantly reduced tumor volume, and the tumor volume significantly increased after the decrease in CRNDE expression compared with the control group (Fig. S1E-G). Mechanically, MGC803 cells can induce autophagy after 24 h of exposure to oxaliplatin (10 μg/m) and 5-FU (400 μg/ml) (Fig. 1e&f). Western blot results showed that in MGC803 cells, CRNDE knockdown could significantly promote autophagy via upregulating the expression of LC3 II and increase the autophagy level induced by oxaliplatin and 5-FU (Fig. 1g, h). Similar results were also found by LC3II and P62 immunohistochemistry in tumor of PDX model (Fig. S1H).

### CRNDE induces proteasome ubiquitination-dependent SRSF6 degradation and contributes to autophagy-induced chemoresistance in GC cells

**Fig. 2:**
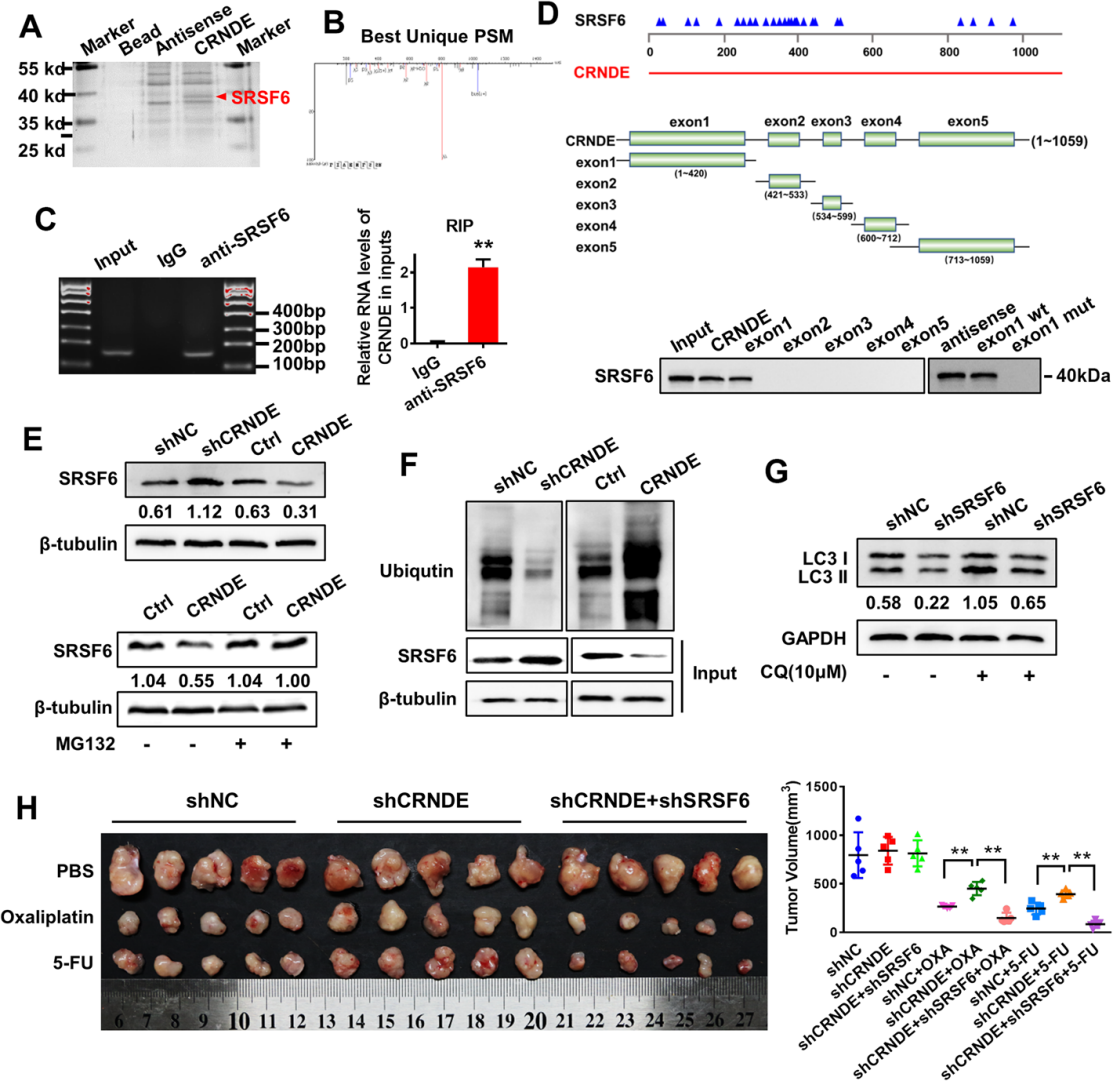
CRNDE induces proteasome ubiquitination-dependent SRSF6 degradation and contributes to autophagy-induced chemoresistance in GC cells. **a** RNA pull-down assay was used to identify the proteins associated with CRNDE. Biotinylated CRNDE and antisense RNA were incubated with cell extracts, and the associated proteins were resolved by SDS-PAGE. The CRNDE-sense-special bands (arrows) were excised and analyzed by mass spectrometry. **b** SRSF6 was identified as CRNDE binding protein by mass spectrometry. **c** RIP assay showed that CRNDE and SRSF6 proteins interact with each other in MGC803 cells. **d** Western blot confirms the presence of SRSF6 in CRNDE pull-down products. **e** The effect of CRNDE on the stability of SRSF6. MG-132 eliminates the effect of CRNDE on the stability of SRSF6. (F) CRNDE affects the ubiquitination of SRSF6 in MGC803 cells. **g** Western blot analyzed the expression of LC3II in MGC803 cells transfected with shSRSF6 plasmid in the presence of CQ. **h** The chemotherapy resistance of MGC803 cells to oxaliplatin and 5-FU caused by interfering with the expression of CRNDE was counteracted after administration of shSFSF6 in vivo. The tumor volume of each group is shown (*n*=5). Representative figures are shown. The results are from three independent experiments. Student’s *t*-test and one-way ANOVA; mean ± SD; the asterisk (**) indicates *P* < 0.01

To identify the proteins that bind to CRNDE, the products obtained from RNA pull-down were separated by SDS-PAGE electrophoresis. After silver staining, we found that there was an obviously different band between CRNDE and its antisense RNA near 40 kD (Fig.2a). Three independent mass spectrometry assays identified SRSF6 as a CRNDE-interacting protein (Fig. 2b). Next, we investigated the interaction of CRNDE and SRSF6 in vivo by RNA-binding protein immunoprecipitation (RIP) experiments. The enrichment of CRNDE was observed by using SRSF6 antibody compared to a nonspecific antibody (IgG control) (Fig. 2c). To identify the interacting domain in CRNDE with SRSF6, we used the ESEfinder website (*http://krainer01.cshl.edu/tools/ESE2/*) to predict the possible binding sites and constructed truncation mutants of each exon of CRNDE. Through an RNA pull-down assay, we found that exon 1 was essential for binding to SRSF6 (Fig. 2d). Then, we evaluated whether CRNDE affected the stability of SRSF6. Western blot analysis showed that the silencing of CRNDE significantly increased SRSF6 protein expression levels, while CRNDE overexpression decreased SRSF6 protein expression levels (Fig. 2e). We used cycloheximide (CHX), a protein synthesis inhibitor, to detect the stability of SRSF6 after interfering with or overexpressing CRNDE. It was found that the stability of the SRSF6 protein increased significantly after CRNDE silencing and decreased significantly after CRNDE overexpression (Fig. S2A). In addition, treatment with the proteasome inhibitor MG-132 restored the stability of SRSF6 protein decreased by CRNDE. These results indicate that the degradation of SRSF6 in gastric cancer cells after CRNDE overexpression is proteasome dependent (Fig. 2e). Moreover, the ubiquitination of SRSF6 increased in MGC803 cells overexpressing CRNDE without MG-132 treatment but decreased in CRNDE knockdown MGC803 cells (Fig. 2f). To explore the role of SRSF6 in GC chemoresistance, we performed interference experiments with plasmids and lentiviruses to inhibit SRSF6 expression. RT-PCR and western blot were used to confirm the transfection efficiencies (Fig. S2B&C). Compared with that of the control group, the sensitivity of MGC803 cells to oxaliplatin and 5-FU was significantly increased by decreasing the expression of SRSF6 in vitro (Fig. 2d) and in vivo (Fig. S3A&B). GSEA (gene set enrichment analysis) showed that SRSF6 was associated with the regulation of cell autophagy (GSE57303; Fig. S2E), suggesting that SRSF6, as a target gene of CRNDE, could participate in the regulation of chemotherapy resistance through autophagy. Subsequently, we explored the effect of SRSF6 on autophagy and found that LC3II expression decreased after silencing SRSF6 expression (Fig. 2g). Rescue experiments showed that transiently transfecting shSRSF6 into CRNDE-knockdown GC cells significantly restored chemotherapy resistance mediated by interfering with CRNDE in vitro (Fig. S3C) and in vivo (Fig. 2h&S2D). These results suggest that SRSF6 is essential for CRNDE-mediated inhibition of GC cell autophagy to promote chemosensitization.

### SRSF6 promotes autophagy activity by regulating alterative splicing of PICALM

**Fig. 3:**
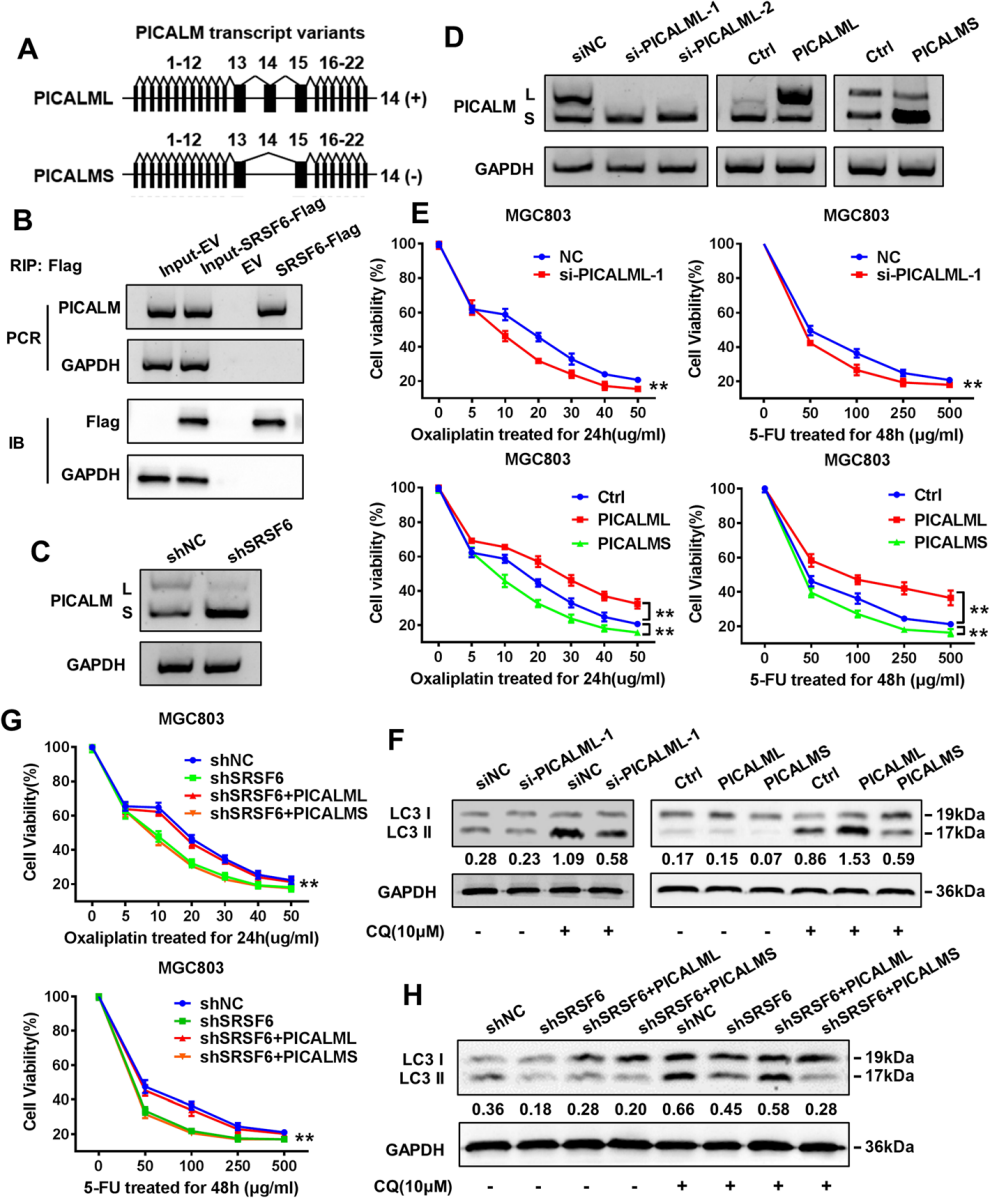
SRSF6 promotes autophagy activity through regulating alterative splicing of PICLAM. **a** Schematic diagram of PICALM splice variants. **b** RIP and RT-PCR for detecting the interaction between SRSF6 protein and PICALM mRNA (upper panels: RT-PCR for PICALM mRNA; lower panels: immunoblotting for SRSF6-flag protein). **c** Depletion of SRSF6 leads to a shift of PICALM splicing module in MGC803 cells. **d** The interference and overexpression of PICLAML and the overexpression efficiency of PICALMS were assessed by western blot. **e** CCK8 assays were used to evaluate the interference and overexpression of PICLAML and overexpression of PICALMS on the sensitivity of MGC803 cells to oxaliplatin and 5-FU. **f** The effect of PICALML and PICALMS on the expression of LC3II in MGC803 cells in the presence of CQ was detected by western blot. **g** PICLAML or PICLAMS were re-introduced into MGC803/shSRSF6 cells for drug sensitivity test. (H) PICLAML and PICLAMS were re-introduced into MGC803/shSRSF6 cells for western blot to detect the expression level of LC3II. Representative figures are shown. The results are from three independent experiments. One-way ANOVA; mean ± SD; the asterisk (**) indicates *P* < 0.01

Considering that SRSF6 is a classical splicing factor, we identified its alternative splicing (AS) targets by next-generation RNA-seq. The results showed that exon skipping (156 events) was more frequent in SRSF6 knockdown cells compared with control cells (Fig. S4). Among these SRSF6-regulated AS events, we found that the PICALM exon 14 skip splice variant was significantly increased after SRSF6 shRNA treatment. The long isoform of human PICALM has 22 exons (encoding PICALML), while the short isoform lacks exon 14 (encoding PICALMS) (Fig. 3a). To further identify whether SRSF6 binds to PICALM mRNA in vivo, we performed RIP assays and found that SRSF6 specifically binds to PICALM RNA (Fig. 3b). Strikingly, when SRSF6 was depleted, a significant L-to-S isoform switch was observed (Fig. 3c). In addition, after the specific depletion of PICALML (Fig. 3d), we observed an increased sensitivity of MGC803 cells to oxaliplatin and 5-FU chemotherapy (Fig. 3e) and decreased expression of LC3II (Fig. 3f). Overexpression of PICALML increased the resistance of MGC803 cells to oxaliplatin and 5-FU and increased the expression of LC3II, while overexpression of PICALMS promoted chemosensitivity and decreased the expression of LC3 II. (Fig. 3d-f). To test whether SRSF6 promotes autophagy through PICALM splicing and thus mediates chemoresistance, we introduced PICALML or PICALMS into SRSF6-deleted GC cells. The reintroduction of PICALML significantly increased the sensitivity of cells to oxaliplatin and 5-FU and decreased the expression of LC3II, while PICALMs had no significant effect (Fig. 3g&h). In general, these results suggest that the function of SRSF6 may depend on the splicing of PICALM.

## Conclusions

Our results provide the basis for the view that the decreased expression of CRNDE in human GC may be of great significance in obtaining chemoresistance, suggesting that CRNDE may be a new prognostic and therapeutic biomarker for GC. In addition, studies on the function and/or mechanism of CRNDE in this report suggest that CRNDE may play a key role in autophagy-mediated chemoresistance by binding to SRSF6 and reducing its protein stability, thereby reducing the selective splicing of PICALM mRNA. When chemotherapy resistance occurs in GC patients during chemotherapy, the effect of chemotherapy may be improved by restoring the expression of CRNDE, which can be verified in clinical practice.

## Supplementary Information

**Additional file 1: Fig. S1**. CRNDE is related to response to 5-FU/oxaliplatin-based chemotherapy in GC patients.

**Additional file 2: Fig. S2**. CRNDE inhibits autophagy-related chemoresistance via inducing SRSF6 degradation in GC cells.

**Additional file 3: Fig. S3**. SRSF6 contributes to CRNDE-induced autophagy activity and chemoresistance in GC cells

**Additional file 4: Fig. S4**. Next-generation RNA-Seq were performed to identify classical splicing factor SRSF6-involved alterative splicing (AS) targets

**Additional file 5.**

## Data Availability

Not applicable.

## Abbreviations

GC: Gastric cancer
LncRNA: Long noncoding RNA
PDX: Patient-derived xenograft
AS: Alternative splicing
FBS: Fetal bovine serum
qRT-PCR: Quantitative RT-PCR
IF: Immunofluorescence
IHC: Immunohistochemistry
