# Supplementary figures and images for: LncRNA CRNDE attenuates chemoresistance in gastric cancer via SRSF6-regulated alternative splicing of PICALM
Source: Mol Cancer. 2021 Jan 4;20:6. doi: 10.1186/s12943-020-01299-y (PMC7780690; doi:10.1186/s12943-020-01299-y)

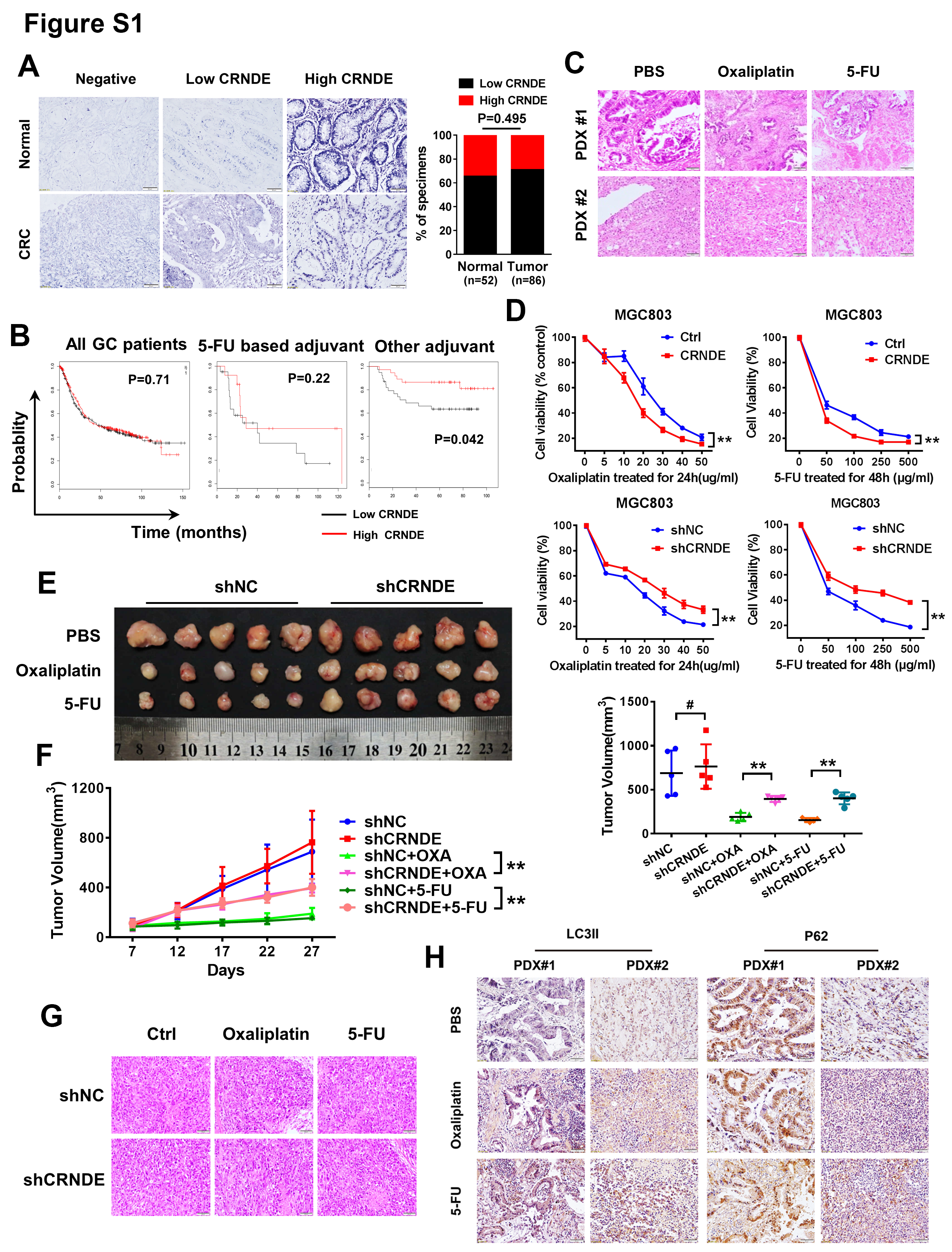

Supplement: Supplementary file 1 — Additional file 1: Fig. S1. CRNDE is related to response to 5-FU/oxaliplatin-based chemotherapy in GC patients. [file 12943_2020_1299_MOESM1_ESM.tif]

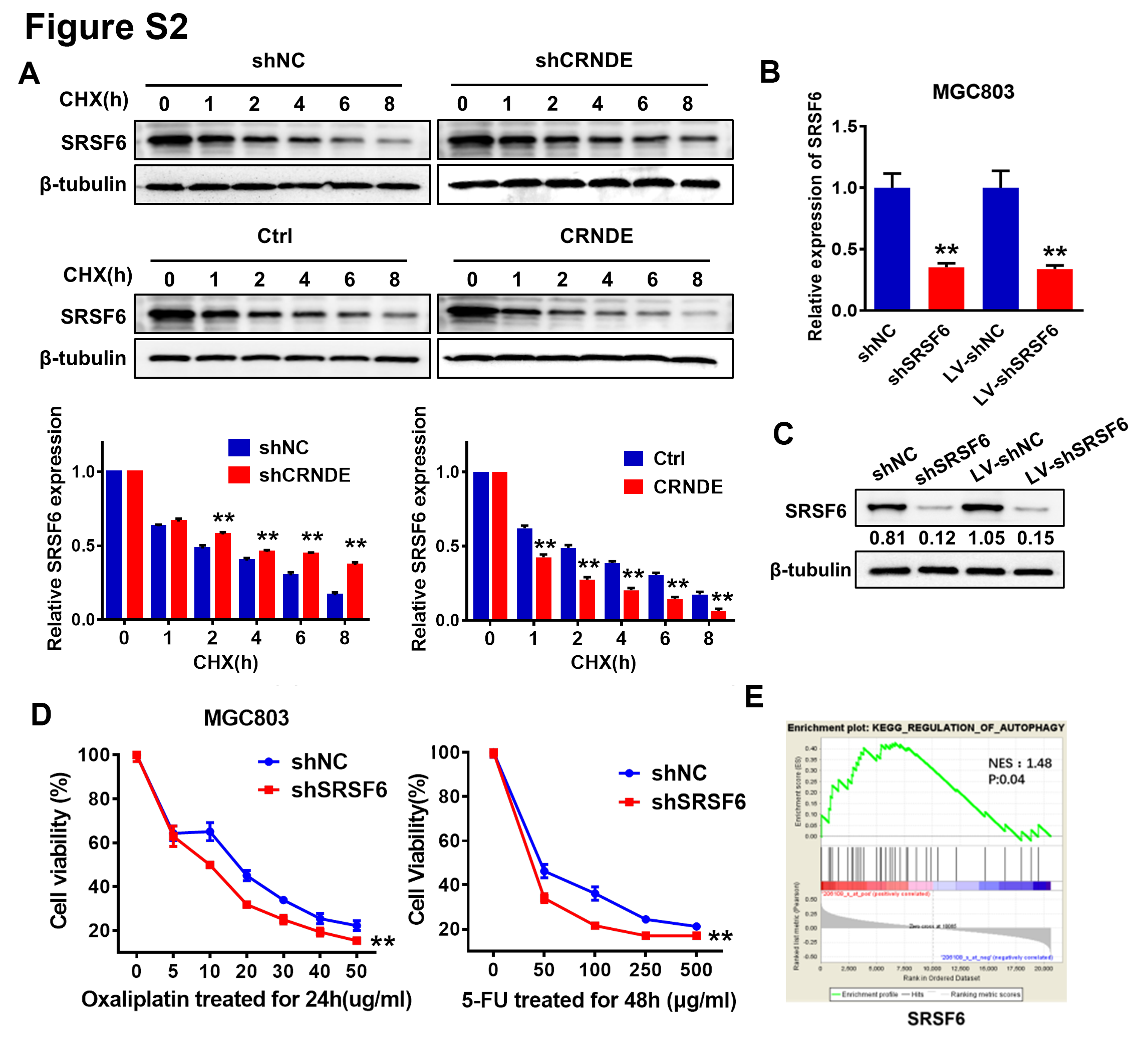

Supplement: Supplementary file 2 — Additional file 2: Fig. S2. CRNDE inhibits autophagy-related chemoresistance via inducing SRSF6 degradation in GC cells. [file 12943_2020_1299_MOESM2_ESM.tif]

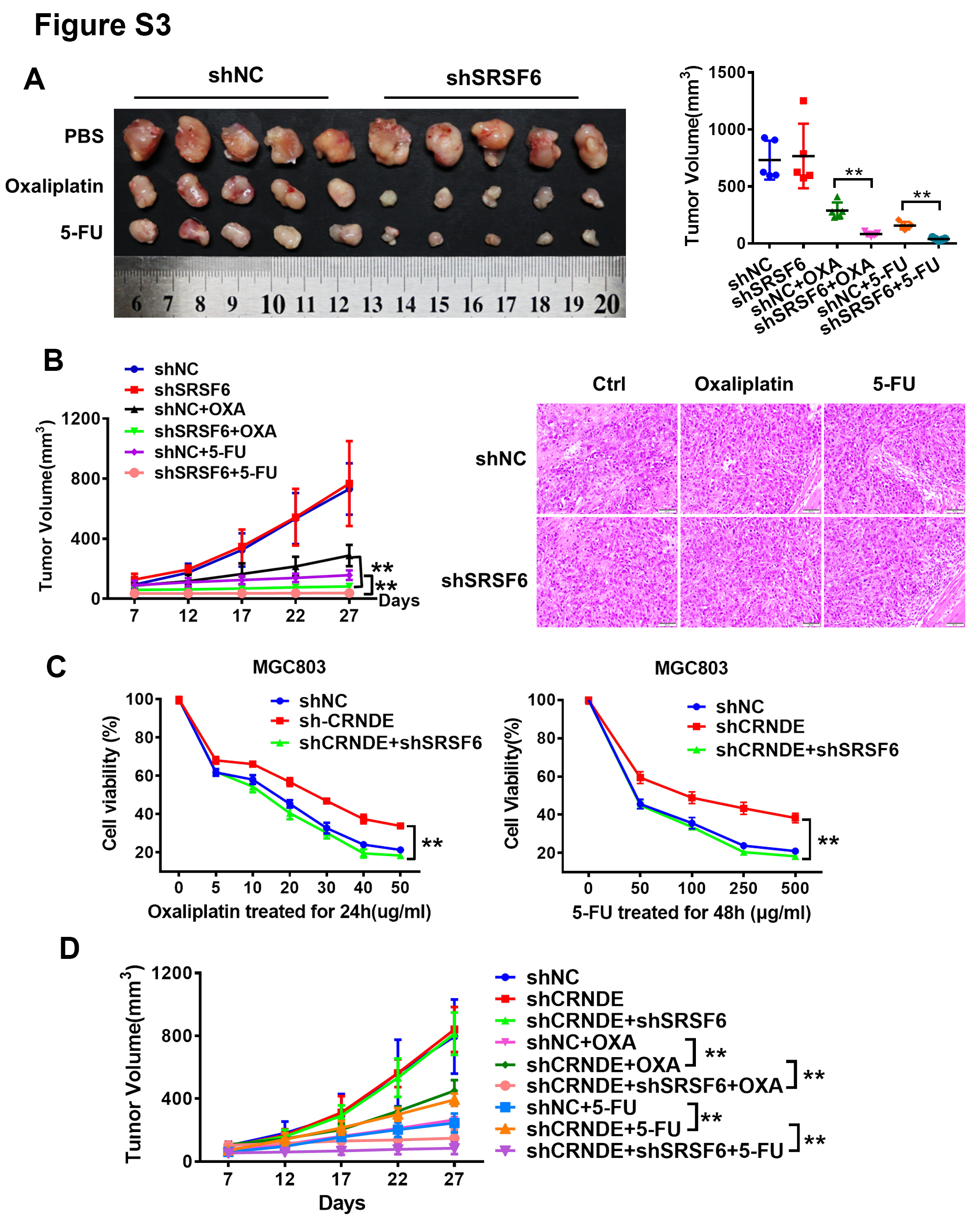

Supplement: Supplementary file 3 — Additional file 3: Fig. S3. SRSF6 contributes to CRNDE-induced autophagy activity and chemoresistance in GC cells [file 12943_2020_1299_MOESM3_ESM.tif]

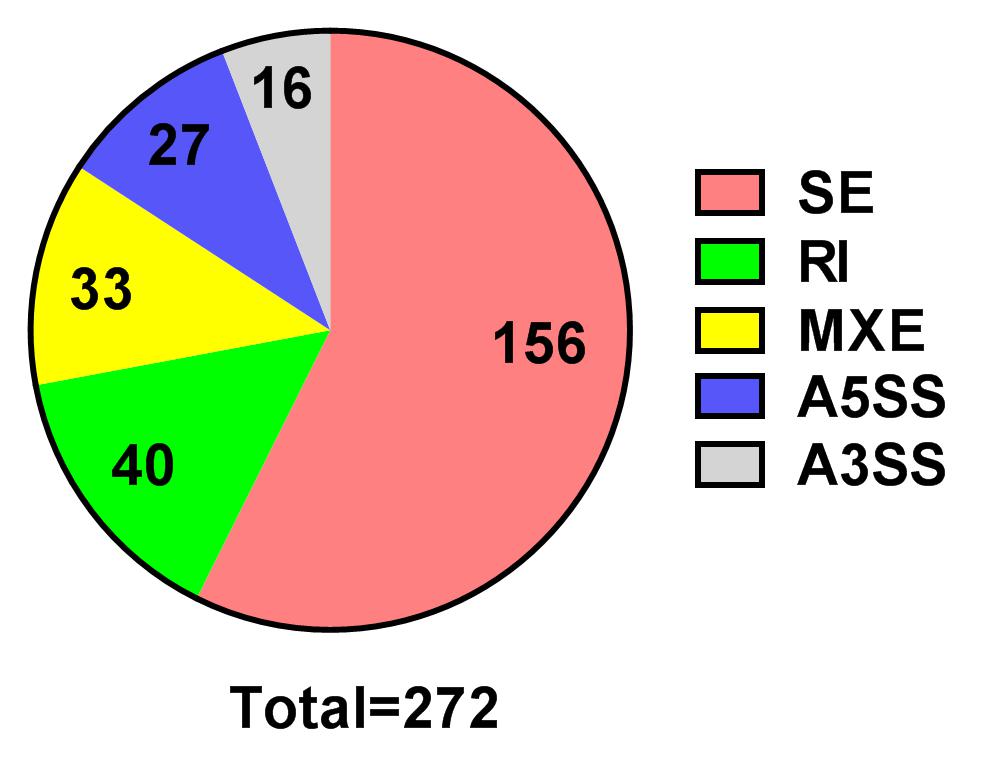

Supplement: Supplementary file 4 — Additional file 4: Fig. S4. Next-generation RNA-Seq were performed to identify classical splicing factor SRSF6-involved alterative splicing (AS) targets [file 12943_2020_1299_MOESM4_ESM.jpg]
