# Supplementary material for: LncRNA CRNDE attenuates chemoresistance in gastric cancer via SRSF6-regulated alternative splicing of PICALM
Source: Mol Cancer. 2021 Jan 4;20:6. doi: 10.1186/s12943-020-01299-y (PMC7780690; doi:10.1186/s12943-020-01299-y)
Supplement: Supplementary file 5 — Additional file 5. [file 12943_2020_1299_MOESM5_ESM.docx]

**Supplementary Methods**

**Cell culture**

The human gastric cancer cell line MGC803 was obtained from the Cell Bank of the Chinese Academy of Sciences (Shanghai, China) and cultured in RPMI-1640 (Gibco, Grand Island, NY, USA) medium containing 10% fetal bovine serum (FBS) (PAA Laboratories, Inc., Pasching, Austria) with 5% CO_2_ at 37°C. Primary gastric cancer cells originate from fresh tissues of gastric cancer patients obtained during surgery at Nanfang Hospital. After the samples are cut into small pieces and digested, they were dissociated into single cell suspensions and cultured in culture flasks. Primary gastric cancer cells were cultured with trypsin and purified manually.

**Overexpression and RNA interference**

Plasmids expressing CRNDE, shCRNDE, SRSF6 and shSRSF6 and all siRNA oligos were purchased from GenePharma (Suzhou, China). GC cells at exponential growth phase were plated into 6-well plates for 24 hours at a density of 0.5 × 10^5^ cells/ml and transfected with 1 mg of siRNA or 4 μg of cDNA using Lipofectamine 2000 reagent for 24 h (Invitrogen; Carlsbad, CA, USA) in reduced serum medium (OPTI-MEM-I; Invitrogen) according to the manufacturer's protocol.

**Lentivirus infection and establishment of stable cell lines**

LV-GFP-RFP-LC3, shCRNDE, shSRSF6 and control vector shNC were purchased from GenePharma (GenePharma, Suzhou, China). Lentiviral particles were used to infect MGC803 or OXA/R and 5-FUR/R cells to establish stable cell lines using puromycin as a selection marker.

**Tumor tissue samples**

Fresh primary gastric cancer tissues were collected from the Department of Gastrointestinal Surgery of Nanfang Hospital. All specimens were frozen in liquid nitrogen. Paraffin-embedded tissues were obtained from Nanfang Hospital as well. Clinicopathological information, including age, sex, pathological stage and TNM stage, was well recorded. This study was approved by the Ethics Committee of Southern Medical University, and all aspects of the study followed the Helsinki Declaration guidelines.

**Establishment of the PDX** **model**

Four-week-old NOD/SCID female mice were purchased from GemPharmatech Co., Ltd., Jiangsu, China. The PDX model was established as previously described. Fresh tumor tissue was cut into small pieces of ~1 mm^3^ and subcutaneously transplanted into the fat pad under the forearm of mice after deep anesthesia induced by isoflurane. After reaching the third generation, when the tumor size reached 50~100 mm^3^, the mice were randomly divided into three groups. Oxaliplatin and 5-FU were injected intraperitoneally every 3 days. The mice were sacrificed 24 days after the treatment or when the tumor volume reached 1500 mm^3^. The researchers were blinded to group allocation in their assessment of the results. All clinical samples were collected after informed consent was obtained from the corresponding patients, and the study was approved by the ethics committee of Nanfang Hospital (Guangzhou, China). All animal experiments involved were conducted under protocols approved by the Southern Medical University Animal Care and Use Committee.

**Western blot assay**

Proteins were extracted by lysis buffer and quantified by a BCA Protein Quantification Kit (KeyGen Biotech, Nanjing, China). The protein lysate was separated by 10% SDS-PAGE and transferred to a PVDF membrane (Roche). Subsequently, the membranes were incubated with specific primary antibodies and corresponding secondary antibodies. The intensity of the protein bands was determined by a Gel-pro analyzer. The densitometry value of the protein was analyzed by ImageJ software. Antibodies against LC3B (NB100-2220; 1:1000; Novus Biologicals, CO, USA), P62 (18420-1-AP; 1:1000; Proteintech, IL, USA), GAPDH (60004-1-Ig; 1:1000; Proteintech, IL, USA), SRSF6 (DF8824; 1:1000; Affinity Biosciences, OH, USA) and β-tubulin (10068-1-AP; 1:500; Proteintech, IL, USA) were used. The experiments were replicated three times.

**RNA pull-down assay**

CRNDE and its antisense RNA were transcribed with T7 RNA polymerase (Roche Diagnostics, USA), biotin labeled with Biotin RNA Labeling Mix (Roche Diagnostics, USA), and treated with DNase I *in vitro*. A total of 50 pmol biotinylated RNA was incubated with magnetic beads (Life Technologies, USA) and mixed with protein. After washing and elution, the retrieved proteins in the pull-down material were stained with silver after SDS-PAGE. The corresponding bands were excised and analyzed by mass spectrometry by a company (Wininnovate Bio, China). The experiments were replicated three times.

**RNA immunoprecipitation**

RNA immunoprecipitation (RIP) experiments were performed with the Magna RIP RNA-Binding Protein Immunoprecipitation Kit (Millipore, MA) according to the manufacturer’s protocol. Anti-SRSF6 antibody (Novus Biologicals, USA; catalog no. NBP2-04142) was used for RIP detection of coprecipitated RNA. The RNA was amplified and detected by RT-PCR. The experiments were replicated three times.

**Nude mouse tumor transplantation model**

Four-week-old BALB / C nude mice were used for oxaliplatin and 5-FU treatment in vivo. The study was approved by the Animal Ethics Committee of Nanfang Hospital. All animal experiments involved were conducted under protocols approved by the Southern Medical University Animal Care and Use Committee. The corresponding cell suspension was subcutaneously injected into the right side of the nude mice (5×10^6^ tumor cells/100 μl PBS per point). The tumor volume was measured with digital caliper and calculated according to the formula: the maximum diameter (L) × minimum tumor diameter (W)^2^/2. When the tumor volume reached 50-100 mm^3^, the mice were randomly divided into control group and treatment group. The dose of oxaliplatin was 10mg/kg mice, and 5-FU 50mg/kg mice were injected intraperitoneally every 3 days. After 3 weeks of treatment, the mice were sacrificed, and the tumor tissue was dissected, fixed with 10% neutral formaldehyde, and embedded in paraffin for further study.

**Real-time PCR (RT-PCR)**

Total RNA was extracted from patient tissues or cultured cells using the Trizol method. Reverse Transcription of RNA to cDNA with PrimerScript^TM^ RT Master Mix (RR036A, Takara, Dalian, China), RT-PCR was performed using TB Green^®^ Premix Ex Taq^TM^ (RR420A, Takara, Dalian, China) according to the instructions. The experiments were replicated for three times.

**Fluorescent confocal microscopy**

OXA/R, 5-FU/R and parental cell MGC803 stably expressing tandem mCherryEGFP-LC3 were seeded and grown in confocal dishes and photographed under confocal microscopy (LSM880, Cari Zeiss, Jena, Germany) 24 hours later. The experiments were replicated for three times.

**CCK8 assays**

Cells were implanted into 96-well plates at a density of 1×10^5^/100μl and incubated for 12 hours. 10μl /well was added to CCK8 reagent (Dojindo, Japan, CK04) and incubated for 2 hours. The absorbance was recoded at 450 nm and calculated the number of viable cells. The experiments were replicated for three times.

**Transmission electron microscopy**

Cells were treated with 2.5% glutaraldehyde containing 0.1 mol/l sodium cacodylate, samples were incubated in 1% osmium tetroxide, dehydrated by increasing concentration gradients of ethanol and acetone. Then embedded in succinate, cut into 50 nm sections, and stained with 3% uranyl acetate and lead citrate. Images were obtained using JEM-1200 TEM (JEOL, Tokyo, Japan). The experiments were replicated for three times.

**ISH and** **IHC staining**

In situ hybridization (ISH) and immunohistochemistry (IHC) staining were performed as previously described (Gao Q. *et al*. Oncogene 2020). Scores were measured by three pathologists: 0 = no staining, 1 = weak staining, 2 = moderate staining, 3 = strong staining. For statistical reasons, the staining index score was divided into two groups: low expression group (0-1) and high expression group (2-3). CRNDE probes (1:1000, MssBio, Guangzhou, China) were used.

**Statistical analysis**

Statistical analysis was carried out with SPSS 20.0 software. For normally distributed data, *t*-test or one-way ANOVA was used for statistics. Data with a non-normal distribution were tested by Dunnett’s test. Survival analysis was performed by the Kaplan-Meier method and by the log-rank test. A P value < 0.05 was considered statistically significant.
